# Analysis of DNA from liquid biopsy: new genetic biomarkers for cancer immunotherapy?

**DOI:** 10.37349/etat.2021.00041

**Published:** 2021-04-30

**Authors:** Carminia Maria Della Corte, Flora Cimmino, Floriana Morgillo

**Affiliations:** 1 Department of Precision Medicine, Oncology, University of Campania “Luigi Vanvitelli”, 80131 Naples, Italy; 2 CEINGE Biotecnologie Avanzate, 80145 Naples, Italy; Istituto Nazionale Tumori-IRCCS-Fondazione G. Pascale, Italy

In last years, introduction of immunotherapeutic agents, such as immune checkpoint inhibitors (ICIs) and chimeric antigen receptor T-cell (CAR-T), are changing the clinical scenario of anti-cancer treatment with great results in multiple cancer types [1]. Especially in thoracic malignancies, including non-small cell lung cancer (NSCLC) and also small cell lung cancer (SCLC) and malignant pleural mesothelioma (MPM), the addition of ICI anti-cytotoxic T-lymphocyte-associated protein 4 (CTLA-4), programmed cell death protein 1 (PD-1), or programmed cell death ligand 1 (PD-L1), are currently used worldwide [2–4].

However, despite the general improvement in patients’ prognosis, only a subgroup of patients achieve a long-term clinical and survival benefit and there is a big gap of knowledge regarding biomarkers of response. Moreover, monitoring anti-cancer immune response in patients maybe not easy, due to the dynamic changes during times, and also preclinical studies on immunotherapy drugs need often specific models, such as syngeneic murine models [5] or *ex vivo* models [6] that preserve immune components, and a great researchers’ expertise.

Until now, among genomic and proteomic biomarkers that have been explored and correlated with immunotherapy response, we can find: high PD-L1 protein expression, tumor mutation burden (TMB) and specific gene mutations associated with DNA mismatch repair deficiency or microsatellite instability [7]. These features can co-exist in cancer and generally scientific community believes that one single biomarker is not sufficient to identify the real “immunotherapy responders”, independently from cancer type [7].

An interesting approach, derived from multiple studies on tumor microenvironment and tumor intrinsic genomic profile, is to define “immune-hot tumors”, characterized by “inflamed phenotype” immune cells infiltrations, that is a surrogated marker of anti-cancer immune response, and by high expression of immune-related proteins and transcripts, including immune checkpoints and cytokines [8]. Interestingly, innate immune response activation, in particular stimulator of interferon signaling pathway (STING), physiologically activated in response to viral infection, is also correlated to immune responsiveness in NSCLC and SCLC [5, 8, 9]. Also, novel combinations, including DNA damaging agents (radiotherapy, chemotherapy, targeted agents, like DNA damage response family genes and monoclonal antibodies) are able to convert otherwise immune-resistant tumors in immune-sensitive tumors, through activation of STING pathway, further proposing there is a big connection between innate and adaptive immune response in cancer patients [5, 8, 9]. For example, clinical data are available on efficacy of combination of anti-PD-1 drug, avelumab, and anti-epidermal growth factor receptor (EGFR), cetuximab, in a small subgroup of patients relapsed NSCLC patients, unselected for any biomarkers, from a proof-of concept study [10]. Interestingly, the proposed mechanism of action of this combination is in the ability of the two antibodies to induce NK-cells mediated antibody dependent cellular cytotoxicity (ADCC) thus involving innate immunity. Moreover, exploratory biomarkers include analysis performed all on “liquid biopsy” (LB) samples, like serial collections of blood samples from enrolled patients. Downregulation of circulating tumor DNA levels (ctDNA), DNA damage response gene mutations and ADCC ability of patients’ derived NK cells were identified as potential biomarkers of response in patients who responded to the experimental combination [10]. Similarly, Chen et al. [11] identified high circulating-free DNA (cfDNA) and specific mutations (such as MIKI67) as predictors of resistance to novel combinations of ICI and anti-angiogenic drugs.

In this clinic scenario, LB represents in our opinion the best method to monitor all these proposed biomarkers in the era of immuno-oncology and to explore novel ones, that can be rapidly transferable to routine clinical practice [12]. LB uses fluids, mainly blood, for biological tests of cfDNA or more specifically ctDNA and circulating tumor cells (CTCs) but also proteins, exosomes and other circulating vesicles [12]. In various cancers, cfDNA/ctDNA quantification at baseline was lower in patients exhibiting superior overall survival and could precede radiographic response in multi-cohort studies [13]. Since ctDNA is easily accessible, genomic sequencing of ctDNA represents a powerful alternative for genetic analysis in patients with NSCLC, particularly when no tissue sample is available. In fact, the European Medicines Agency (EMA) and Food and Drug Administration (FDA) have approved the use of ctDNA for EGFR mutation assessment prior to tyrosine kinase inhibitor (TKI) treatment. Promising cfDNA sequencing results for TMB assessment and copy number instability are being studied for the initial stratification of NSCLC patients eligible for immunotherapy [14]. Blood TMB (bTMB) assessment represents an extensive use of cfDNA analysis in patients whose tissue material is scarce or of limited quality for the TMB test [15]. Furthermore, serial monitoring of ctDNA analysis could serve as a non-invasive strategy to predict clinical benefit and long-term survival for ICI-treated patients [14] and novel combinations including ICI, as cited above [10, 11]. Moreover, genome-wide sequencing of ctDNA can be used to detect dynamic change in genome instability to monitor response to immunotherapy [14] and targeted cfDNA deep sequencing can detect clinically actionable mutations and how they evolve under pressure of ICI. Interestingly, several ctDNA mutations in serine/threonine kinase 11 (STK11) have been strongly associated with immunotherapy-treated unresponsive NSCLC patients and validation assays are currently underway [8, 15]. Previous studies [7] have found that a small percentage of patients benefiting from the PD-1/PD-L1 inhibitor show no PD-L1 expression in tumor tissues. Although the spatiotemporal heterogeneity of PD-L1 may influence PD-L1 as predictive biomarkers, quantification of PD-L1 in CTCs can be a complementary diagnostic tool for deciding whether ICI therapy is appropriate [16]. Recently, PD-L1 single nucleotide polymorphisms (SNPs) have been thought to increase PD-L1 expression suggesting that *PD-L1* gene variants may also be a biomarker for patient stratification [17]. In view of the heterogeneity of the tumor and the variability of PD-L1 expression, we believe that the use of the source of blood samples from cancer patients to evaluate both the molecular characterizations of PD-L1 in CTCs [18] and the characteristics of germline DNA of PD-L1 [19], will open completely new scenarios for associating PD-L1 expression with clinicopathological factors of NSCLC.

Thus, large prospective clinical trials are needed to provide evidence for the use of ctDNA in the clinical setting. We foresee that applications of LB and generally the use of liquid samples from cancer patients for biomarkers test can rapidly evolve and adapt to novel emerging data from research for and personalize therapies with ICI for cancer patients.

## Abbreviations

CAR-T: chimeric antigen receptor T-cell
cfDNA: circulating-free DNA
CTCs: circulating tumor cells
ctDNA: circulating tumor DNA
ICI: immune checkpoint inhibitor
LB: liquid biopsy
NSCLC: non-small cell lung cancer
PD-1: programmed cell death protein 1
PD-L1: programmed cell death ligand 1
SCLC: small cell lung cancer
STING: stimulator of INTerferon signaling pathway
TMB: tumor mutation burden

## Declarations

### Copyright

© The Author(s) 2021.
